# Supplementary figures and images for: Towards Understanding Non-Infectious Growth-Rate Retardation in Growing Pigs
Source: Proteomes. 2019 Sep 11;7(3):31. doi: 10.3390/proteomes7030031 (PMC6789591; doi:10.3390/proteomes7030031)

Supplementary material figure S1

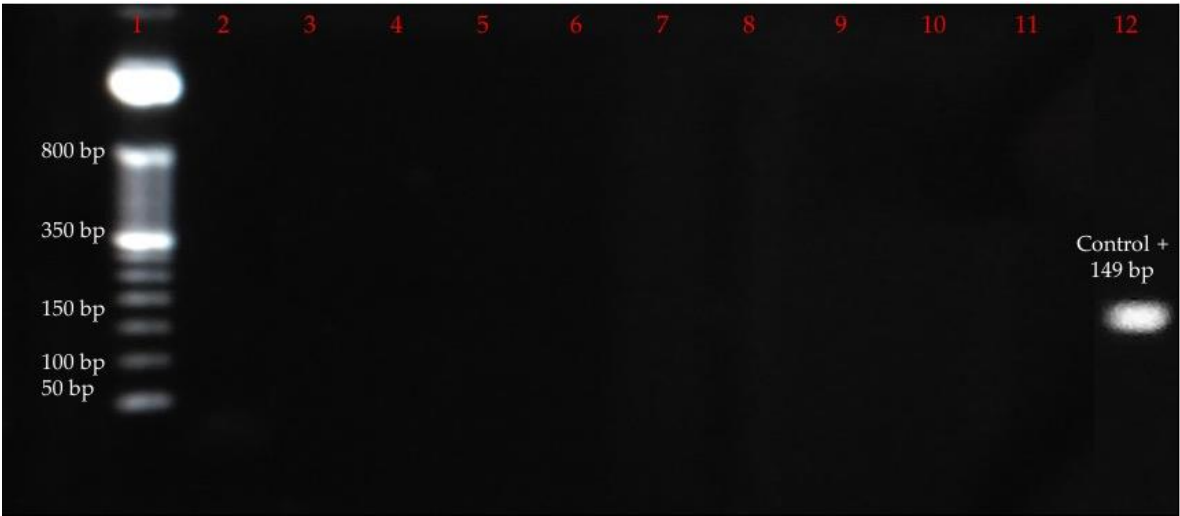

Supplementary material figure S2

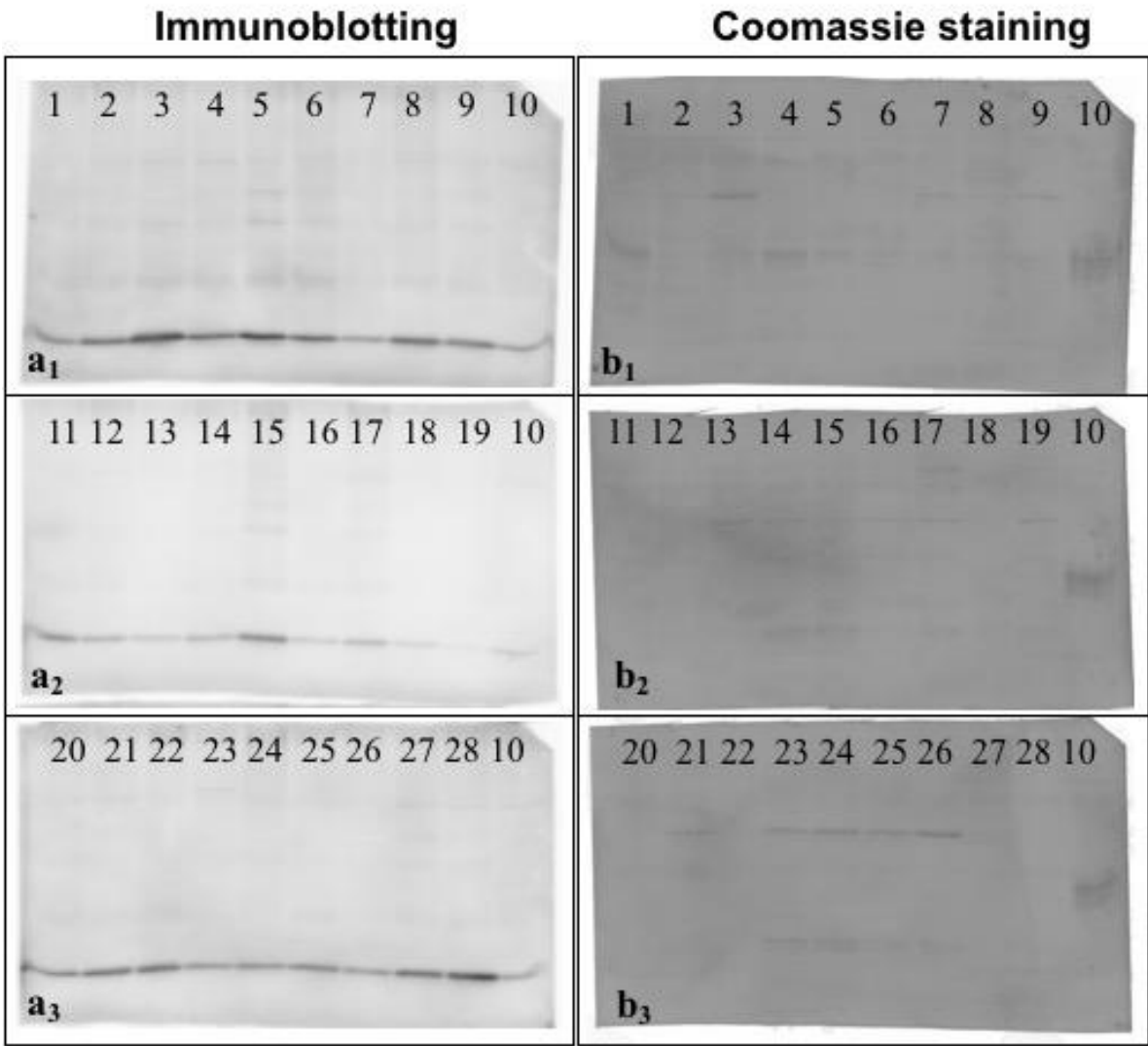

Supplement: Supplementary file 1 [file proteomes-07-00031-s001.zip › proteomes-549317-supplementary/proteomes-549317-supplementary.pdf]
